# Associations of Early Parental Concerns and Feeding Behaviors with Child’s Diet Quality through Mid-Childhood

**DOI:** 10.3390/nu12113231

**Published:** 2020-10-22

**Authors:** Véronique Gingras, Karen M. Switkowski, Sheryl L. Rifas-Shiman, Sabrina Faleschini, Emily Oken, Marie-France Hivert

**Affiliations:** 1 Division of Chronic Disease Research across the Lifecourse, Department of Population Medicine, Harvard Medical School and Harvard Pilgrim Health Care Institute, Boston, MA 02215, USA; veronique_gingras@harvardpilgrim.org (V.G.); karen_switkowski@harvardpilgrim.org (K.M.S.); sheryl_rifas@harvardpilgrim.org (S.L.R.-S.); sabrina.faleschini.1@ulaval.ca (S.F.); emily_oken@harvardpilgrim.org (E.O.); 2 School of Psychology, Laval University, Quebec City, QC G1V0A6, Canada; 3 Department of Nutrition, Harvard TH Chan School of Public Health, Boston, MA 02115, USA; 4 Diabetes Unit, Massachusetts General Hospital, Boston, MA 02114, USA

**Keywords:** parental practices, weight concerns, restrictive feeding, pressure to eat, diet quality, child

## Abstract

Parental feeding practices have been associated with children’s dietary intakes, yet the directionality of these associations remains unclear. Among 1172 mother-child pairs from Project Viva, we aimed to examine associations of parental concerns and feeding behaviors at 2 years (behaviors dichotomized as yes vs. no), with diet quality (Youth Healthy Eating Index; YHEI) in early (mean 3.2, SD 0.3 years; *n* = 1076) and mid-childhood (mean 7.8, SD 0.7 years; *n* = 993). We used multivariable linear regression models adjusted for sociodemographic characteristics, parental body mass index (BMI), maternal diet quality in pregnancy, and child’s BMI z-score and diet quality at 2 years. Early parental concerns about their child becoming overweight (15%) was associated with lower YHEI (*β* −1.54 points; 95%CI −2.75, −0.33; fully adjusted model) in early childhood. Early parental concerns about their child becoming underweight (7%) was associated with lower YHEI (−2.19 points; −4.31, −0.07) in early childhood, but the association was attenuated after adjustment for child’s BMI z-score and diet quality at 2 years. We did not find associations of parental restrictive feeding (8%) and parental pressure to eat (47%) with child’s YHEI through mid-childhood. In conclusion, we found no evidence that early parental concerns and feeding behaviors independently contribute to child’s diet quality through childhood.

## 1. Introduction

Healthy eating during childhood is crucial to ensure proper growth and development, and to maintain a healthy body weight and optimal health across the lifespan [1]. Current American dietary guidelines suggest a healthy diet for children including a variety of fruits and vegetables, whole grains, low-fat and fat free dairy, protein foods, and oils, but limiting saturated fats and trans fats, added sugars, and sodium [2]. Dietary indices have been developed to assess and monitor dietary quality in populations. For example, the Healthy Eating Index was developed by the United States (US) Department of Agriculture (USDA) to monitor changes in dietary intake over time in the US population [3], and this score has then been adapted for children (Youth Healthy Eating Index; YHEI) [4]. In the US, dietary quality in children and adolescents has been improving through recent decades [5]. Yet, the overall diet quality remains low in US children and it decreases with advancing age [5,6]. Moreover, important disparities in diet quality across sociodemographic groups persist [6].

Dietary habits tend to track from childhood to adulthood [7,8,9]; thus, establishing healthy dietary patterns early in childhood could result in healthier diet throughout the life course and reduced long-term risk of diseases. The social environment and family are key players in shaping dietary habits and lifestyle behaviors in children. Parents in particular have been shown to play an important role in influencing children’s food preferences and eating patterns through their own preferences, beliefs and feeding practices [10]. Parental influences early in life may have long lasting consequences shaping the child’s eating habits and later obesity risk [11]. Parent-child feeding relationships have been long recognized as complex, as both parties interact and engage in food selection, quantities of food ingested, and regulation behaviors [12]. In addition to what types of foods parents provide their children, how parents feed their children is likely to have long-lasting consequences. Parental feeding practices have been described as behaviors or strategies employed by parents in order to influence their child’s diet or food intake, for example, pressuring their child to eat more or restricting their child’s intakes [13,14]. These parental feeding practices have been associated with children’s dietary intakes and body weight status [15,16]. However, findings are not consistent across studies, including the directionality and magnitude of the effects [15,17,18,19]. Moreover, most prior studies were cross-sectional or did not include a prospective follow-up. Assessment of diet quality over multiple time points during childhood is needed to investigate potential long-term effects of parental feeding practices. Previous studies do not allow for examining whether early life parental feeding practices have a lasting measurable influence on diet quality.

In this study, we aimed to examine associations of parental feeding behaviors and weight-related concerns assessed when the child was 2 years old with the child’s dietary quality in early (~3 years) and mid-childhood (~8 years) in a large, well-characterized prospective study of mother-child pairs. We hypothesized that factors associated with parental feeding restriction and pressure to eat would be associated with poorer child diet quality in later childhood, whereas parental weight concerns would be associated with better child diet quality.

## 2. Materials and Methods

We studied participants from Project Viva, a prospective pre-birth cohort. Women included in this cohort were recruited between 1999 and 2003 during their first prenatal visit at one of the offices of what is now Atrius Harvard Vanguard Medical Associates in Eastern Massachusetts, US. To be included, women needed to understand English, be at <22 weeks of pregnancy, and have a singleton pregnancy. Full inclusion and exclusion criteria, as well as recruitment methods, have been published [20] and all study instruments are publicly available online at www.hms.harvard.edu/viva/. The study is also registered in clinicaltrials.gov as NCT02820402. Briefly, the study was established to examine associations of pre- and postnatal factors with maternal and child health, and includes maternal data collected in-person during pregnancy, maternal and child data collected during the delivery admission and during infancy, and every 3 to 4 years subsequently. Additional information was also collected yearly between in-person visits using questionnaires, and data was collected from medical records. The study was approved by the Institutional Review Board of Harvard Pilgrim Health Care (IRB reference number 235301-155—Project Viva: A longitudinal study of health for the next generation; latest approval date 17 October 2019). All participants provided written informed consent at enrollment and each postnatal follow-up visit, and all procedures were in accordance with the ethical standards for human experimentation established by the Declaration of Helsinki.

After obtaining informed consent, we conducted in-person visits with women during their first trimester of pregnancy and at mid-pregnancy (26–28 weeks of gestation). Information was also collected from medical records at delivery and through childhood. Mothers and children then completed in-person visits in the hospital at birth, in infancy (6 months), early (mean age, 3.2 years old) and mid-childhood (mean age, 7.9 years old). Beginning at 1 year postpartum, we mailed annual questionnaires to mothers.

From the original 2128 children, we excluded a total of 956 children because of missing exposure (no report of parental feeding behaviors or weight concerns at 2 years of age; *n* = 840), no measured outcomes (no report of dietary intakes in early or mid-childhood; *n* = 94) or birth <34 weeks of gestation (*n* = 22). Compared to the 1172 participants included in this analysis, those excluded had mothers that were younger (mean age 31.0 vs. 32.5 years old), had a greater pre-pregnancy body mass index (BMI; mean 25.4 vs. 24.5 kg/m^2^), and lower dietary quality during pregnancy (mean Alternate Healthy Eating Index (A-HEI) score 59.6 vs. 61.2 points), were less likely to be married or cohabitating at the infancy visit (88% vs. 94%), and were less likely to have a college degree (53% vs. 74%) and to have an annual household income > US dollars (USD) 70,000 (49% vs. 65%). In addition, children excluded from this analysis were more likely to be non-white (45% vs. 29%). We found no differences between participants included compared to excluded for child’s sex and paternal BMI.

### 2.1. Exposures

The exposures were maternal reports of concerns about child’s weight (overweight and underweight) and use of restrictive feeding practices or pressure to eat. The exposures were measured with modified questions from the Child Feeding Questionnaire (CFQ) [13] completed by mothers when the child was approximately 2 years old (mean 2.1; SD 0.0 years). This questionnaire assesses parental beliefs, attitudes, and practices regarding child feeding and is validated in children from 2–11 years [13]. We included in our 2-year questionnaire several questions derived from the CFQ that assessed child feeding as well as parental concerns. The first domain, restrictive feeding, was assessed with the question “I have to be careful not to feed my child too much” and for this analysis we dichotomized the answer as yes (strongly agree or agree) or no (strongly disagree or disagree). The second domain, pressure to eat, was assessed with the following questions: “I often have to encourage my child to eat more;” “I have to be sure that my child finishes the bottle;” “I have to be sure that my child finishes everything in the cup;” “I have to be sure that my child finishes all the food on the plate.” For this analysis, we dichotomized answers as yes (strongly agree or agree) or no (strongly disagree or disagree), and categorized mothers who answered yes to at least one of these questions as pressuring their child to eat more. Modifications to the feeding behaviors questions and answer format were made originally to include them in the longitudinal cohort, beginning at 6 months of age. Although not all questions from the CFQ were included, we used confirmatory factor analysis to test a 2-factor hypothetical structure of parental feeding behaviors, including restrictive feeding and pressure to eat, and described details elsewhere [21,22]. For sensitivity analyses, as described below, we also dichotomized the answers for pressure to eat as yes (strongly agree) or no (strongly disagree, disagree or agree), and categorized mothers who answered yes to at least one of the four questions as pressuring their child to eat more. Finally, we included two questions regarding concerns about child’s weight, assessed using yes/no questions; “Are you worried that your child will become underweight as he/she is growing up?” and “Are you worried that your child will become overweight as he/she is growing up?”.

### 2.2. Outcomes

Outcomes include overall dietary quality (primary outcome) and specific dietary group intakes (secondary outcomes) assessed in early childhood (mean 3.2; SD 0.3 years) and mid-childhood (mean 7.8; SD 0.7 years).

At the early childhood in-person visit, mothers completed a 87-item food frequency questionnaire (FFQ), validated for use in preschool-age children [23]. Frequency of intake of specific foods and drinks during the previous month was assessed and average food intakes were derived. Response categories included, for foods: “Never,” “Less than once per week,” “Once per week,” “Nearly daily or daily,” and “2 or more time per day,” and for drinks and snack foods: “Never,” “Less than once per week,” “Once per week,” “2–4 times per week,” “Nearly daily or daily,” “2–4 times per day”, and “5 or more times per day”. Child’s daily intakes of fruits and vegetables, red and processed meats, milk, sugar-sweetened beverages, fruit juice, snack foods/baked products, and fast food were derived using reported intakes. Snack foods/baked products included chips, cookies or brownies, cake or cupcake, pie, crackers, Jell-O, donuts, muffins, chocolate candy, and other candy. Fast food intake was defined by the question “In the past month, on average, how often did your child eat something from a fast-food restaurant (e.g., McDonald’s, Burger King, Taco Bell, etc.)?”

We then derived the YHEI [4] total score from the FFQ data. In total, 10 out of 13 of the original score components were available for the total score calculation, which ranged from 0 to 85 points, with higher scores indicating a healthier diet (Table S1). The components included were; whole grains, vegetables, fruits, dairy, meat ratio (lean meat, fish, and legumes: red and processed meat), snack foods, soda and drinks, multivitamin use, and fast food outside home. The components not available in our 3-year questionnaire were consumption of visible animal fat, eating breakfast, and eating dinner with family; these components have been shown to add little variability in the calculation of the total score [4].

At the mid-childhood in-person visit, mothers reported children’s dietary intakes using the Prime-Screen, a dietary screener composed of 18 items asking about frequency of consumption of specific food groups, including fruits and vegetables, processed meat, dairy products, baked products, sugar-sweetened beverages and fast food [24]. Using validation data collected in adults, the average correlation of intake estimates from the Prime Screen with estimates obtained from a full FFQ over 18 food groups was 0.6 [24]. We derived the mid-childhood YHEI score from the information obtained from the Prime-Screen, including the same ten out of the thirteen original components [4] as for our early childhood YHEI calculation (Table S1).

### 2.3. Covariates

During the enrollment visit, women reported their age, education level, marital status, household income, height, and pre-pregnancy weight, from which we calculated pre-pregnancy BMI (kg/m^2^). We dichotomized educational status as college graduate vs. not a college graduate, annual household income as > vs. ≤ USD 70,000 and marital status as married/cohabiting vs. other. Women also reported paternal weight and height, from which we derived paternal BMI. Child’s sex was extracted from hospital medical records at birth. At the early childhood visit, mothers reported the child’s race/ethnicity, which we categorized as non-Hispanic white, black, Hispanic, or other. We calculated maternal dietary quality during pregnancy using a modified version of the Alternate Healthy Eating Index (A-HEI) [25,26] from validated self-administered semi-quantitative FFQ adapted for pregnancy and completed during the first and second trimesters of pregnancy [27,28]. At 2 years, mothers completed the same 87-item food frequency questionnaire (FFQ) as the one used for the early childhood visit [23]. We derived the 2-year YHEI total score using the same previously described calculation (Table S1). Finally, we calculated BMI (weight in kilograms divided by length or height in meters squared) using data on weight and length/height obtained from medical records where pediatric clinics recorded these measurements at each well-child visit. Measurement methods have been previously described [29] and we derived age- and sex-specific BMI z-scores based on the WHO Child Growth Standards [30]. We defined 2-year BMI z-score as BMI z-score measurement >12 months and <24 months of age, and if more than one value was available, we used the one closest to 18 months.

### 2.4. Statistical Analyses

We computed the mean (SD) or number (%) for maternal, parental/household, and child’s baseline characteristics stratified by parental weight-related concerns and feeding behaviors (yes vs. no) and assessed differences between parents who have concerns or use the defined behaviors or not using simple linear or logistic regression analyses (Table 1). We also computed the mean (SD) for overall dietary intakes as well as diet quality score at early and mid-childhood as presented in Table 2.

We assessed associations of parental weight-related concerns and feeding behaviors with child’s overall diet quality and specific dietary components in early childhood (Table 3) and mid-childhood (Table 4) using multivariable linear regression models. Model 1 was adjusted for child’s sex and age at outcome assessment and for sociodemographic characteristics, including maternal age at inclusion, education, and marital status, as well as annual household income and child’s race/ethnicity. Model 2 was additionally adjusted for maternal diet quality during pregnancy and pre-pregnancy BMI as well as paternal BMI. Finally, to examine the influence of child’s baseline characteristics in our associations, model 3 was additionally adjusted for child’s BMI z-score between 12–24 months and diet quality at 2 years.

Since parental pressure to eat at 2 years (defined as “agree” or “strongly agree” to at least one of the four questions) was relatively common in our cohort (47%), we conducted sensitivity analyses using a more restrictive categorization of the exposure; only mothers who answered “strongly agree” to at least one of the four previously listed questions were categorized as pressuring their child to eat, which resulted in the exposure being observed in 10% of parents. In this sensitivity analysis, we repeated our multivariable linear regression models for the associations of parental pressure to eat at 2 years with this more restrictive categorization and child’s overall diet quality and specific dietary components in early and mid-childhood (Tables S2 and S3).

We used multiple imputation to impute missing data on covariates. All study variables were included in the imputation model and results are based on pooled results of 50 imputed datasets. We conducted all analyses using SAS version 9.4 (SAS Institute, Cary, NC, USA).

## 3. Results

Table 1 presents maternal, parental/household and child’s baseline characteristics stratified by parental weight-related concerns and feeding behaviors. When their child was 2 years old, 15% of parents were concerned about their child becoming overweight and 7% about their child becoming underweight, 47% pressured their child to eat and 8% restricted their intakes. Mothers included in the present study were (mean (SD)) 32.5 (4.8) years old at inclusion with a pre-pregnancy BMI of 24.5 (5.0) kg/m^2^. Most participants were college educated (74.1%), married (94.4%), and with an annual household income over USD 70,000 (65.0%), whereas children were mostly white (70.9%). As shown in Table 1, several sociodemographic characteristics differences by parental feeding concerns and practices were found. Mainly, maternal pre-pregnancy BMI and child’s BMI at 2 years were associated with both parental feeding behaviors and weight-related concerns, and child’s race/ethnicity was associated with both feeding behaviors as well as concerns about the child becoming underweight. Other associations of sociodemographic characteristics and parental feeding behaviors and concerns are presented in Table 1.

We present overall dietary intakes as well as diet quality score at early and mid-childhood in Table 2. Overall diet quality (YHEI total score, out of 85 points) was lower in mid-childhood (mean total score 49.9; SD 10.2 points) compared to early childhood (mean score 55.3; SD 9.6 points).

As shown in Table 3, after adjusting for sociodemographic characteristics, parental BMI and maternal diet quality during pregnancy as well as child BMI z-score and YHEI total score at 2 years, parental concern at 2 years about their child becoming overweight was associated with lower YHEI total score in early childhood (*β* −1.54 points; 95%CI −2.75, −0.30). Early concern about their child becoming overweight was additionally associated with greater child’s intake of red and processed meat in early childhood (*β* 0.14 serving/day; 95%CI 0.05, 0.24; fully adjusted model). Parental concern about the child becoming underweight was associated with lower YHEI total score (*β* −2.19 points; 95%CI −4.31, −0.07) in early childhood after adjusting for sociodemographic characteristics, parental BMI and maternal diet quality during pregnancy. However, after further adjustment for child’s BMI z-score and YHEI total score at 2 years, the association was attenuated (*β* −1.63 points; 95%CI −3.36, 0.10).

Parental pressure to eat and restrictive feeding at 2 years were not associated with child’s YHEI total score in early childhood. However, early parental pressure to eat was associated with higher child’s fruit juice (*β* 0.19 serving/day; 95%CI 0.03, 0.36; fully adjusted model) intake in early childhood. It was also associated with snack food intakes (*β* 0.12 serving/day; 95%CI 0.01, 0.22; Model 2) in early childhood, although this association was attenuated after further adjustment for child’s BMI z-score and YHEI total score at 2 years (*β* 0.09 serving/day; 95%CI −0.01, 0.20). In our sensitivity analysis (Table S2) looking at parental pressure to eat at 2 years using a more stringent categorization of the exposure (strongly agree; 10.4% of participants), parental pressure to eat was inversely associated with child’s total YHEI total score (*β* −1.47 points; 95%CI −2.86, −0.08) and positively associated with snack foods/baked products intake (*β* 0.24 serving/day; 95%CI 0.07, 0.42) in fully adjusted models in early childhood. Finally, parental restrictive feeding was associated with lower child sugar-sweetened beverages intake (*β* −0.17 serving/day; 95%CI −0.28, −0.06; fully adjusted model).

As shown in Table 4, none of the parental concerns or behaviors at 2 years were associated with YHEI total score in mid-childhood and none of the associations with specific dietary components observed in early childhood persisted into mid-childhood. Early concern about their child becoming overweight was associated with higher child’s intake of baked products (*β* 0.07 serving/day; 95%CI 0.01, 0.13; fully adjusted model) in mid-childhood while early concern about their child becoming underweight was associated with none of the food group intakes in mid-childhood. Parental restrictive feeding at 2 years was associated with child’s lower dairy intakes (*β* −0.31 serving/day; 95%CI −0.59, −0.04; fully adjusted model) and early parental pressure to eat was associated with none of the food group intakes in mid-childhood. In our sensitivity analysis with a more stringent categorization of parental pressure to eat, the association with YHEI total score in mid-childhood was largely attenuated as compared to early childhood and no longer significant, and we found an association with fast food intake in mid-childhood (*β* 0.20 serving/day; 95%CI 0.04, 0.36; fully adjusted model).

## 4. Discussion

In this prospective analysis using data collected through mid-childhood, we found modest associations of parental weight-related concerns with child’s later dietary quality. Parental concerns at 2 years about their child becoming overweight was associated with lower diet quality in early childhood, but this association did not persist in mid-childhood. We also found an association between early parental concern about their child becoming underweight with poorer diet quality in early childhood although this association was mitigated by inclusion of child’s BMI z-score and diet quality at 2 years. Associations with specific dietary components were mostly inconsistent throughout childhood, with modest effect sizes.

### 4.1. Parental Concerns—Overweight

In our study, parental concerns about their child becoming overweight was associated with poorer diet quality in early childhood, independently of child’s BMI z-score at 2 years. The association did not persist in mid-childhood. Parents awareness regarding their child’s overweight risk would have ideally resulted in preventive actions to reduce this risk, such as offering healthier foods to their child. However, studies have shown that greater concern about child’s overweight risk was associated with unfavorable parental strategies [31,32] or with a lack of action to prevent weight gain in their children [33]. Developing practical healthy tools for parents might be useful to improve their child’s diet in relation to such concerns.

### 4.2. Parental Concerns—Underweight

Parental concerns about their child becoming underweight was also associated with poorer diet quality in early childhood; however, inclusion of BMI z-score and diet quality at 2 years in our model mitigated that association. This finding suggests a possible reverse causality; parents’ concerns might be a response to prior child’s eating habits and body weight. Food offerings at 3 years are under parental control, and it is possible that parents offer more foods of lesser quality to their child in order to promote greater overall intakes in reaction to the perceived inadequacy of their child’s weight or in response to their child’s pickiness. It is also possible that parental concerns do not equate with behavior change capacity. Nonetheless, these findings suggest a potential need for education among parents who perceive their child as at risk of becoming underweight to ensure parents offer healthier food choices to their child.

### 4.3. Parental Pressure to Eat

Our findings on associations of parental pressure to eat at 2 years and higher child’s intakes of fruit juice and snack foods in early childhood are consistent with some of the previous studies [34,35]. In contrast, pressure to eat was associated with healthier dietary intakes, such as greater fruit and vegetable intakes [36,37] and lower consumption of snack foods [38] in previous cross-sectional studies, whereas other studies showed no association of parental pressure to eat and examined dietary intakes in children [39,40]. Only a few longitudinal studies assessed the association of parental pressure to eat and child’s later dietary intakes. In one study, parental pressure to eat assessed through infancy was associated with lower diet quality at 3 years, independently of several maternal characteristics, including BMI and sociodemographics, not accounting for child baseline characteristics except gender [41]. In a second study, pressure to eat assessed at 6 years was associated with unhealthy dietary behaviors (snacking and sugar-sweetened beverages consumption) at 8 years, although the associations were attenuated when accounting for child dietary behaviors and BMI z-score at age 6 [42]. Our study adds to previous findings by having included two timepoints, early and mid-childhood, and by having considered in our analyses both parental characteristics, including both parents’ BMI and maternal diet during pregnancy, as well as child characteristics, including BMI z-score and diet quality at baseline. In early childhood, parents make the choices regarding the foods that are offered, while in mid-childhood the child is more likely to have some control over what he eats, and none of the associations observed in early childhood persisted into mid-childhood, suggesting no lasting effect of early parental feeding behaviors in our cohort. In our sensitivity analysis looking at this exposure with a more restrictive categorization (Tables S2 and S3), we found additional associations of parental pressure to eat at 2 years with lower YHEI total score and higher snack foods intake in early childhood, suggesting potential different effects for children from parents who reported a stronger agreement in performing this behavior. Parental pressure to eat might represent a potential target for family-based interventions aiming to improve children’s diet, however longer-term longitudinal studies remain necessary to assess whether parental pressure to eat may have long-lasting consequences on child’s eating behaviors. Also, understanding the underlying reasons for parental use of pressure to eat could help clarify the relationship between this behavior and child’s diet. Pressure to eat is possibly used by parents to promote greater food intakes if they fear their child might not consume sufficient quantities of food or specific nutrients. Webber et al. showed that parents pressure their child to eat in response to concerns about inadequate intakes or because they perceive their child as too thin [43]. A review of qualitative studies also suggested that parental pressure to eat is associated with fussy eating in children, an association that is likely to be bi-directional [44].

### 4.4. Parental Restrictive Feeding

For parental restrictive feeding, we did not find associations with later overall diet quality, but it was associated with lower intakes of sugar-sweetened beverages at early childhood and with lower dairy intakes at mid-childhood. A review by Loth KA published in 2016 reported that exposure to parental restrictive feeding is associated with higher child’s intakes of sugar-sweetened beverages, palatable snack foods, and calorie-dense foods [45], and a systematic review published in 2017 concluded that parental restrictive feeding is associated with higher child’s snack intakes [46]. However, findings vary according to populations studied, and similarly to pressure to eat, associations of parental restrictive feeding and dietary intakes in children have mostly been studied cross-sectionally. One possible explanation for the associations observed with parental restrictive feeding could be an increased desire by children to consume foods previously prohibited or restricted once it is made available [47]. The discrepancy between our findings and previous studies could be related to the study design, the length of follow-up, or it could be related to the parental report of dietary intakes, i.e., parents who restricted their child’s intakes in early life might be less likely to report such intakes in their children later. Also, we did not assess which specific foods were restricted by parents in our study, and it is possible that overall restrictive feeding practices do not have the same influence over child’s intakes as the restriction of specific types of foods.

Strengths of this study include the longitudinal assessment of diet quality at two time points in childhood in nearly 1200 children from Project Viva. By including diet quality and dietary intakes at 8 years in this analysis, we are more likely to have captured the child’s own preferences and choice of food intakes, although we acknowledge that parents probably still have at least some control over the child’s food choices. Another strength of this study is that our analyses were adjusted for several parental and child characteristics that we found to be associated with feeding practices, including socioeconomic characteristics, child’s sex and race/ethnicity, maternal diet, and BMI of both parents. Socioeconomic context appears to influence parental feeding practices, and these characteristics are important to consider when trying to understand the relationship between parental feeding practices and child’s dietary quality. Despite further adjustment for child’s BMI z-score and YHEI at 2 years, reverse causality remains possible as parents’ feeding practices may be an adaptation to child’s previous dietary habits or body weight trajectories. Moreover, collecting data for such a large prospective study sometimes require compromises to limit the participants’ burden when assessing numerous aspects of child’s development over several decades. In our cohort, we used questions derived from the CFQ to assess parental restrictive feeding and pressure to eat, yet we modified the questions and response categories to accommodate their use for children at a very young age. Thus, not all original domains were assessed. These changes need to be considered when comparing findings from this study with other published results. In addition, we had numerous secondary outcomes (specific dietary components) and looked at associations with four different exposures, and we acknowledge these findings could be less robust due to multiple comparisons. Finally, both parental feeding practices and child’s dietary intakes were reported by mothers, which could have introduced bias, and we acknowledge that generalizability of our findings is limited considering the relatively high socioeconomic status of our study sample.

## 5. Conclusions

We found that early-life parental concerns and feeding behaviors may have a modest contribution to child’s dietary intakes throughout childhood; however, we did not observe associations with overall diet quality in both early and mid-childhood. Our study does not provide evidence that parental feeding practices in early life are associated with child diet quality in later years.

Overall, relationships between child’s characteristics, parental feeding practices and children’s dietary behaviors are complex, and well-characterized longitudinal studies remain needed to better understand these relationships and long-term implications. Studies including a longitudinal follow-up and using a more refined assessment of feeding practices and styles in early life remain needed to better understand whether these exposures have long-term effects on child’s eating behaviors; as yet, tools are currently limited to assess a more extensive array of parental beliefs and practices, and their development could help better understand how children’s dietary behaviors develop under specific conditions.

## Figures and Tables

**Table 1:** Parental, household, and child characteristics by parental feeding concerns and practices at 2 years among participants from Project Viva (Mean (SD) or %).

	Overall	Concerns about Child Weight—Overweight		Concerns about Child Weight—Underweight		Pressure to Eat		Restrictive Feeding	
		Yes *n* = 178 (15%)	No *n* = 992 (85%)	Yes *n* = 77 (7%)	No *n* = 1094 (93%)	Yes *n* = 545 (47%)	No *n* = 617 (47%)	Yes *n* = 98 (8%)	No *n* = 1069 (92%)
Characteristics									
Maternal									
Age at enrollment, years	32.5 (4.8)	32.7 (4.5)	32.5 (4.8)	32.6 (4.6)	32.5 (4.8)	32.0 (5.0) ^a^	32.9 (4.6)	31.6 (5.7)	32.6 (4.7)
Pre-pregnancy BMI, kg/m^2^	24.5 (5.0)	27.0 (6.2) ^a^	24.0 (4.6)	23.4 (4.7) ^a^	24.6 (5.0)	24.2 (4.9) ^a^	24.8 (5.1)	26.5 (6.2) ^a^	24.3 (4.8)
Education, college graduate	74.1%	80.3% ^a^	73.1%	75.3%	74.0%	75.6%	72.9%	55.1% ^a^	76.1%
Marital status, married or cohabitating	94.4%	96.1%	94.1%	98.7%	94.1%	93.4%	95.3%	84.7% ^a^	95.2%
Diet score (A-HEI) during pregnancy	61.2 (9.8)	60.4 (9.8)	61.3 (9.8)	62.0 (10.0)	61.1 (9.8)	61.2 (9.8)	61.1 (9.9)	59.5 (10.1)	61.3 (9.8)
Paternal/household									
Household income, >70,000 US dollars	65.0%	66.3%	64.8%	61.5%	65.3%	64.4%	65.8%	37.7% ^a^	67.8%
Paternal BMI, kg/m^2^	26.4 (3.8)	28.0 (4.5) ^a^	26.0 (3.6)	25.5 (3.4)	26.4 (3.9)	26.2 (3.9)	26.5 (3.7)	27.1 (4.6) ^a^	26.3 (3.7)
Child									
Sex, female	49.7%	57.3% ^a^	48.2%	48.1%	49.7%	45.7% ^a^	53.5%	46.9%	50.0%
Race/ethnicity									
White	70.9%	71.9%	70.7%	61.0% ^a^	71.6%	66.2% ^a^	74.9%	37.8% ^a^	74.0%
Black	11.3%	7.9%	11.9%	14.3%	11.1%	11.2%	11.2%	33.7%	9.2%
Hispanic	3.1%	3.9%	2.9%	5.2%	2.9%	4.4%	1.9%	10.2%	2.4%
Other	14.8%	16.3%	14.5%	19.5%	14.4%	18.2%	12.0%	18.4%	14.4%
Body mass index, z-score at 2 years	0.71 (1.03)	1.06 (1.03) ^a^	0.64 (1.01)	0.11 (1.03) ^a^	0.75 (1.02)	0.48 (0.99) ^a^	0.91 (1.03)	1.04 (1.06) ^a^	0.68 (1.02)
YHEI total score at 2 years	55.4 (9.4)	55.4 (9.0)	55.4 (9.4)	54.3 (10.4)	55.5 (9.3)	54.8 (9.6) ^a^	55.9 (9.1)	54.2 (9.7)	55.5 (9.3)

^a^ Significantly different (*p* < 0.05) compared to the group with the absence of the corresponding parental concern or behavior.

**Table 2:** Dietary intakes in early and mid-childhood among participants from Project Viva included in this analysis.

Diet Quality and Food Groups	*n*	Mean (SD)	Interquartile Range
Early childhood			
Youth-Healthy Eating Index, score out of 85 points	1051	55.3 (9.6)	49.1–62.2
Fruits and vegetables, serving/day	1074	4.2 (2.1)	2.7–5.4
Red and processed meats, serving/day	1054	0.8 (0.6)	0.4–11
Milk, serving/day	1071	2.2 (1.3)	1.0–3.0
Sugar-sweetened beverages, serving/day	1072	0.2 (0.5)	0–0.2
Fruit juice, serving/day	1070	1.7 (1.4)	0.9–3.0
Snack foods/baked products, serving/day	1050	1.6 (0.9)	1.0–2.1
Fast food, serving/week	1073	0.6 (0.6)	0–1.0
Mid-Childhood			
Youth-Healthy Eating Index, score out of 85 points	971	49.9 (10.2)	51.4–66.2
Fruits and vegetables, serving/day	993	2.8 (1.5)	0.5–1.0
Processed meat, serving/day	993	0.5 (0.4)	0.2–0.6
Dairy products, serving/day	993	2.1 (1.2)	1.1–2.9
Baked products, serving/day	993	0.4 (0.3)	0.1–0.4
Sugar-sweetened beverages, serving/day	986	0.3 (0.4)	0–0.4
Fast food, serving/week	991	0.7 (0.8)	0.5–1.0

**Table 3:** Adjusted ^a^ linear regression coefficients (*β* (95% CI)) for associations of parental feeding concerns and behaviors at 2 years and child’s diet quality (reference group is the absence of concerns or of the behavior) and food groups intakes at early childhood (mean 3.2, SD 0.3 years) among participants from Project Viva.

		Concerns about Child Weight—Overweight	Concerns about Child Weight—Underweight	Pressure to Eat	Restrictive Feeding
		Yes	Yes	Yes	Yes
Food groups	Model	*β* (95% CI)			
Youth Healthy Eating Index (YHEI), total points	1	−2.00 (−3.58, −0.43)	−2.01 (−4.33, 0.32)	−0.51 (−1.67, 0.65)	0.93 (−1.28, 3.15)
	2	−1.26 (−2.76, 0.24)	−2.19 (−4.31, −0.07)	−0.61 (−1.67, 0.46)	1.30 (−0.73, 3.33)
	3	−1.54 (−2.75, −0.33)	−1.63 (−3.36, 0.10)	−0.16 (−1.02, 0.71)	0.75 (−0.88, 2.39)
Fruits and vegetables, serving/day	1	−0.09 (−0.44, 0.27)	−0.04 (−0.56, 0.47)	−0.15 (−0.41, 0.11)	0.31 (−0.18, 0.81)
	2	−0.07 (−0.42, 0.28)	−0.02 (−0.52, 0.47)	−0.15 (−0.40, 0.10)	0.34 (−0.14, 0.81)
	3	−0.11 (−0.44, 0.22)	0.07 (−0.40, 0.53)	−0.06 (−0.30, 0.17)	0.23 (−0.21, 0.68)
Red and processed meat, serving/day	1	0.15 (0.06, 0.25)	0.08 (−0.05, 0.22)	0.01 (−0.06, 0.08)	0.02 (−0.11, 0.15)
	2	0.14 (0.04, 0.23)	0.09 (−0.05, 0.22)	0.01 (−0.06, 0.08)	0.01 (−0.12, 0.14)
	3	0.14 (0.05, 0.24)	0.08 (−0.05, 0.22)	0.00 (−0.06, 0.07)	0.02 (−0.11, 0.15)
Milk, serving/day	1	−0.02 (−0.22, 0.19)	−0.12 (−0.42, 0.19)	0.09 (−0.06, 0.24)	−0.15 (−0.44, 0.14)
	2	0.01 (−0.21, 0.22)	−0.12 (−0.43, 0.18)	0.09 (−0.07, 0.24)	−0.14 (−0.43, 0.15)
	3	−0.01 (−0.22, 0.21)	−0.09 (−0.40, 0.22)	0.11 (−0.04, 0.27)	−0.17 (−0.46, 0.12)
Sugar-sweetened beverages, serving/day	1	0.02 (−0.06, 0.09)	−0.01 (−0.12, 0.11)	−0.05 (−0.11, 0.01)	−0.16 (−0.27, −0.05)
	2	0.01 (−0.07, 0.09)	−0.01 (−0.12, 0.10)	−0.05 (−0.11, 0.01)	−0.17 (−0.27, −0.06)
	3	0.01 (−0.07, 0.08)	0.01 (−0.11, 0.12)	−0.05 (−0.10, 0.01)	−0.17 (−0.28, −0.06)
Fruit juice, serving/day	1	0.01 (−0.21, 0.24)	0.00 (−0.32, 0.33)	0.18 (0.01, 0.34)	−0.10 (−0.41, 0.22)
	2	0.02 (−0.22, 0.25)	0.00 (−0.32, 0.33)	0.17 (0.01, 0.34)	−0.10 (−0.42, 0.21)
	3	0.01 (−0.23, 0.24)	0.02 (−0.31, 0.35)	0.19 (0.03, 0.36)	−0.11 (−0.43, 0.21)
Snack foods/baked products, serving/day	1	0.10 (−0.05, 0.25)	0.18 (−0.03, 0.40)	0.11 (−0.00, 0.22)	−0.12 (−0.33, 0.09)
	2	0.06 (−0.09, 0.22)	0.20 (−0.02, 0.42)	0.12 (0.01, 0.22)	−0.13 (−0.34, 0.08)
	3	0.07 (−0.08, 0.22)	0.18 (−0.03, 0.39)	0.09 (−0.01, 0.20)	−0.10 (−0.31, 0.11)
Fast food, serving/week	1	0.03 (−0.07, 0.14)	−0.03 (−0.18, 0.13)	0.07 (−0.01, 0.14)	−0.07 (−0.22, 0.07)
	2	−0.04 (−0.15, 0.07)	−0.01 (−0.16, 0.14)	0.07 (−0.00, 0.15)	−0.10 (−0.24, 0.05)
	3	−0.04 (−0.15, 0.06)	−0.01 (−0.16, 0.14)	0.07 (−0.01, 0.14)	−0.09 (−0.23, 0.05)

^a^ Model 1: adjusted for child’s sex and age at outcome assessment, maternal education, marital status and age at inclusion, household income, and child’s race/ethnicity; Model 2: Model 1 additionally adjusted for maternal pre-pregnancy body mass index, paternal body mass index and maternal diet quality during pregnancy; Model 3: Model 2 additionally adjusted for child body mass index z-score from and YHEI at 2 years.

**Table 4:** Adjusted ^a^ linear regression coefficients (*β* (95% CI)) for associations of parental feeding concerns and behaviors at 2 years (reference group is the absence of concerns or of the behavior) and child’s diet quality and food groups intakes at mid-childhood (mean 7.8, SD 0.7 years) among participants from Project Viva.

		Concerns about Child Weight—Overweight	Concerns about Child Weight—Underweight	Pressure to Eat	Restrictive Feeding
		Yes	Yes	Yes	Yes
Food groups	Model	*β* (95% CI)			
Youth Healthy Eating Index (YHEI), total points	1	−0.94 (−2.67, 0.80)	−0.48 (−2.96, 1.99)	−0.92 (−2.19, 0.35)	0.54 (−1.76, 2.85)
	2	−0.10 (−1.77, 1.57)	−0.56 (−2.87, 1.74)	−1.09 (−2.27, 0.09)	0.75 (−1.40, 2.90)
	3	−0.21 (−1.79, 1.38)	−0.21 (−2.41, 2.00)	−0.60 (−1.73, 0.54)	0.59 (−1.44, 2.62)
Fruits and vegetables, serving/day	1	−0.23 (−0.49, 0.04)	0.22 (−0.16, 0.60)	−0.14 (−0.33, 0.05)	0.17 (−0.18, 0.52)
	2	−0.16 (−0.42, 0.11)	0.22 (−0.15, 0.58)	−0.16 (−0.34, 0.03)	0.19 (−0.15, 0.53)
	3	−0.16 (−0.42, 0.10)	0.25 (−0.12, 0.61)	−0.13 (−0.31, 0.06)	0.18 (−0.15, 0.51)
Processed meat, serving/day	1	0.03 (−0.05, 0.10)	−0.01 (−0.12, 0.09)	0.04 (−0.01, 0.09)	−0.07 (−0.17, 0.02)
	2	0.03 (−0.05, 0.10)	−0.02 (−0.12, 0.09)	0.04 (−0.01, 0.10)	−0.07 (−0.17, 0.02)
	3	0.03 (−0.05, 0.10)	−0.02 (−0.12, 0.09)	0.04 (−0.01, 0.10)	−0.07 (−0.17, 0.03)
Dairy products, serving/day	1	0.01 (−0.20, 0.22)	0.12 (−0.18, 0.42)	0.08 (−0.07, 0.24)	−0.29 (−0.57, −0.02)
	2	0.05 (−0.17, 0.26)	0.11 (−0.19, 0.41)	0.08 (−0.07, 0.23)	−0.29 (−0.56, −0.02)
	3	0.03 (−0.19, 0.24)	0.16 (−0.15, 0.46)	0.10 (−0.05, 0.26)	−0.31 (−0.59, −0.04)
Baked products, serving/day	1	0.06 (0.01, 0.12)	0.07 (−0.02, 0.15)	0.03 (−0.02, 0.07)	0.01 (−0.07, 0.08)
	2	0.07 (0.01, 0.13)	0.07 (−0.02, 0.15)	0.03 (−0.02, 0.07)	0.01 (−0.07, 0.08)
	3	0.07 (0.01, 0.13)	0.07 (−0.02, 0.15)	0.02 (−0.02, 0.07)	0.01 (−0.07, 0.09)
Sugar-sweetened beverages, serving/day	1	0.02 (−0.04, 0.08)	0.04 (−0.05, 0.12)	0.02 (−0.02, 0.07)	−0.02 (−0.10, 0.06)
	2	0.01 (−0.05, 0.07)	0.04 (−0.05, 0.12)	0.03 (−0.02, 0.07)	−0.02 (−0.10, 0.05)
	3	0.01 (−0.05, 0.07)	0.03 (−0.05, 0.12)	0.02 (−0.02, 0.06)	−0.02 (−0.10, 0.06)
Fast food, serving/week	1	0.01 (−0.13, 0.15)	0.07 (−0.12, 0.27)	0.04 (−0.07, 0.14)	−0.00 (−0.19, 0.18)
	2	−0.06 (−0.20, 0.08)	0.09 (−0.10, 0.29)	0.04 (−0.06, 0.14)	−0.02 (−0.20, 0.16)
	3	−0.07 (−0.21, 0.07)	0.10 (−0.10, 0.30)	0.04 (−0.06, 0.15)	−0.03 (−0.21, 0.15)

^a^ Model 1: adjusted for child’s sex and age at outcome assessment, maternal education, marital status and age at inclusion, household income, and child’s race/ethnicity; Model 2: Model 1 additionally adjusted for maternal pre-pregnancy body mass index, paternal body mass index, and maternal diet quality during pregnancy; Model 3: Model 2 additionally adjusted for child body mass index z-score from and YHEI at 2 years.

## Supplementary Materials

The following are available online at https://www.mdpi.com/2072-6643/12/11/3231/s1, Table S1: Calculation of the Youth Healthy Eating Index (Y-HEI) components 1–10 in Project Viva from the 2-year, 3-year and 7-year old food frequency questionnaires, total score range 0–85, Table S2: Adjusted linear regression coefficients (β (95% Confidence Interval (CI))) for associations of pressure to eat (strongly agree vs. other answer categories) at 2 years (reference group is the absence of pressure to eat) and child’s diet quality and food groups intakes at early childhood (mean 3.2, SD 0.3 years) in participants from Project Viva, Table S3: Adjusted linear regression coefficients (β (95% Confidence Interval (CI)))) for associations of pressure to eat (strongly agree vs. other answer categories) at 2 years (reference group is the absence of pressure to eat) and child’s diet quality and food groups intakes at mid-childhood (mean 7.8, SD 0.7 years) in participants from Project Viva.
