# Supplementary material for: Associations of Early Parental Concerns and Feeding Behaviors with Child’s Diet Quality through Mid-Childhood
Source: Nutrients. 2020 Oct 22;12(11):3231. doi: 10.3390/nu12113231 (PMC7690442; doi:10.3390/nu12113231)
Supplement: Supplementary file 1 [file nutrients-12-03231-s001.pdf]

Table S1. Calculation of the Youth Healthy Eating Index (YHEI) components 1-10 in Project Viva from the 2-year, 3-year and 7-year old food frequency questionnaires[a], total score range 0 – 85.

#	Components	Maximum Score	Standard for Maximum Score	Minimum Score	Standard for Minimum Score	Included Variables
1	Whole grains	10	≥2	0	0	Brown rice; dark bread; hot cereal
2	Vegetables	10	≥3	0	0	Broccoli; cabbage, coleslaw, or cauliflower; carrots; corn; green beans; lettuce salad; potatoes (baked, boiled or mashed); mixed vegetables; peas (canned, frozen or fresh); peppers (green, red, or hot); spinach; squash (orange or winter); tomatoes; vegetable soup; sweet potatoes or yams
3	Fruits	10	≥3	0	0	Apple or apple sauce; banana; strawberries or other berries; cantaloupe; orange or grapefruit; grapes; watermelon; orange juice; other 100% juice; peach or plum; pear; raisins or prunes
4	Dairy	10	≥3	0	0	Cheese; cottage cheese; cream cheese; ice cream; yogurt; milk, including chocolate milk
5	Meat ratio	10	≥2	0	0	Numerator: baked beans or chili beans; other dried beans, dried peas or lima beans; peanut butter; fried chicken, chicken nuggets; other chicken or turkey; canned tuna; fried fish, fish sticks; other fish; tofu or soybeans; eggs; nuts<br>Denominator: beef – steak or roast;

						hamburger, meatballs, or meatloaf; cold cuts (baloney, salami, ham); ham - baked or steak; hot dogs; pork - chops, roast, or ribs; sausage; bacon
6	Snack foods	10	0	0	≥3	Biscuit, cake or cupcake; chips (potato, corn or others); cookies or brownies; crackers, donut or fried dough; sweet roll or muffin; pie; chocolate candy; other candy; Jello; pudding
7	Soda and drinks	10	0	0	≥3	Fruit drinks (Hi-C, Kool-Aid, lemonade); hot chocolate; soda; sugar-free soda
8	Multivitamin use	5	Daily	0	Never	Chewable multivitamins; multivitamin drops
9	Margarine and butter	5	Never	0	≥2 pats/day	Butter; stick margarine; tub margarine
10	Fast foods outside home	5	Never	0	Daily	N/A

[a] Some food groups included in the 7-year questionnaire differed slightly from the 2 and 3-year questionnaires; for vegetables, the questionnaire included dark green leafy vegetables, broccoli, cauliflower, cabbage, brussels sprouts, carrots and other vegetables; for fruits, it included citrus fruits and other fruits; for meat ratio, the numerator included fish/seafood and the denominator included beef, pork or lamb and processed meats; for snack foods, donuts, cookies, muffins, crackers, cakes, sweet rolls, and pastries were included; for dairy, cream cheese was not included in the questionnaire; and for margarine and butter, only margarine was included.

Table S2. Adjusted[a] linear regression coefficients (β (95% Confidence Interval (CI))) for associations of pressure to eat (strongly agree vs. other answer categories) at 2 years (reference group is the absence of pressure to eat) and child's diet quality and food groups intakes at early childhood (mean 3.2, SD 0.3 years) in participants from Project Viva.

		**Pressure to eat**
		Yes, (n = 120; 10.4% of total study sample)[b]
Food groups	Model	β (95% CI)
Youth Healthy Eating Index (YHEI), points	1	-2.67 (-4.56, -0.78)
	2	-2.27 (-4.00, -0.54)
	3	-1.47 (-2.86, -0.08)
Fruits and vegetables, serving/day	1	-0.48 (-0.90, -0.05)
	2	-0.38 (-0.79, 0.03)
	3	-0.23 (-0.62, 0.15)
	1	-0.04 (-0.15, 0.08)

Red and processed meat, serving/day	2	-0.04 (-0.15, 0.07)
	3	-0.06 (-0.17, 0.05)
Milk, serving/day	1	-0.06 (-0.31, 0.19)
	2	-0.05 (-0.30, 0.20)
	3	-0.02 (-0.27, 0.23)
Sugar-sweetened beverages, serving/day	1	-0.04 (-0.14, 0.05)
	2	-0.05 (-0.14, 0.04)
	3	-0.05 (-0.14, 0.04)
Fruit juice, serving/day	1	0.17 (-0.10, 0.43)
	2	0.17 (-0.10, 0.44)
	3	0.19 (-0.09, 0.46)
Snack foods / baked products, serving/day	1	0.28 (0.10, 0.46)
	2	0.28 (0.11, 0.46)
	3	0.24 (0.07, 0.42)
Fast food, serving/week	1	0.00 (-0.12, 0.13)
	2	0.01 (-0.12, 0.13)
	3	-0.01 (-0.13, 0.11)

[a] Model 1: adjusted for child's sex and age at outcome assessment, maternal education, marital status and age at inclusion, household income and child's race/ethnicity; Model 2: Model 1 additionally adjusted for maternal pre-pregnancy body mass index, paternal body mass index and maternal diet quality during pregnancy; Model 3: Model 2 additionally adjusted for child body mass index z-score from and YHEI at 2 years. [b] Outcomes (food groups) were available for 1050 – 1074 participants at early childhood.

Table S3. Adjusted[a] linear regression coefficients (β (95% Confidence Interval (CI)))) for associations of pressure to eat (strongly agree vs. other answer categories) at 2 years (reference group is the absence of pressure to eat) and child's diet quality and food groups intakes at mid-childhood (mean 7.8, SD 0.7 years) in participants from Project Viva.

		**Pressure to eat**
		Yes, (n = 120; 10.4% of total study sample)[b]
Food groups	Model	β (95% CI)
Youth Healthy Eating Index (HEI), point	1	-1.65 (-3.63, 0.33)
	2	-1.60 (-3.45, 0.25)
	3	-1.00 (-2.76, 0.77)
Fruits and vegetables, serving/day	1	-0.03 (-0.33, 0.28)
	2	-0.03 (-0.32, 0.27)
	3	0.02 (-0.27, 0.31)
Processed meat, serving/day	1	0.05 (-0.03, 0.14)
	2	0.05 (-0.04, 0.13)
	3	0.04 (-0.04, 0.13)
Dairy products, serving/day	1	0.05 (-0.19, 0.29)
	2	0.05 (-0.19, 0.29)
	3	0.07 (-0.17, 0.32)
Baked products, serving/day	1	0.07 (0.00, 0.14)
	2	0.07 (-0.00, 0.13)
	3	0.07 (-0.00, 0.13)
Sugar-sweetened beverages, serving/day	1	0.01 (-0.06, 0.08)
	2	0.01 (-0.06, 0.07)
	3	-0.00 (-0.07, 0.06)
Fast food, serving/week	1	0.18 (0.03, 0.34)
	2	0.20 (0.04, 0.35)
	3	0.20 (0.04, 0.36)

[a] Model 1: adjusted for child's sex and age at outcome assessment, maternal education, marital status and age at inclusion, household income and child's race/ethnicity; Model 2: Model 1 additionally adjusted for maternal pre-pregnancy body mass index, paternal body mass index and maternal diet quality during pregnancy; Model 3: Model 2 additionally adjusted for child body mass index z-score from and YHEI at 2 years. [b] Outcomes (food groups) were available for 971 – 993 participants at early childhood.
